# Simultaneous selection of multiple important single nucleotide polymorphisms in familial genome wide association studies data

**DOI:** 10.1038/s41598-023-35379-y

**Published:** 2023-05-25

**Authors:** Subhabrata Majumdar, Saonli Basu, Matt McGue, Snigdhansu Chatterjee

**Affiliations:** 1 University of Minnesota Twin Cities, Minneapolis, USA; 2 Present Address: AI Risk and Vulnerability Alliance, Seattle, USA

**Keywords:** Statistics, Genome-wide association studies

## Abstract

We propose a resampling-based fast variable selection technique for detecting relevant single nucleotide polymorphisms (SNP) in a multi-marker mixed effect model. Due to computational complexity, current practice primarily involves testing the effect of one SNP at a time, commonly termed as ‘single SNP association analysis’. Joint modeling of genetic variants within a gene or pathway may have better power to detect associated genetic variants, especially the ones with weak effects. In this paper, we propose a computationally efficient model selection approach—based on the e-values framework—for single SNP detection in families while utilizing information on multiple SNPs simultaneously. To overcome computational bottleneck of traditional model selection methods, our method trains one single model, and utilizes a fast and scalable bootstrap procedure. We illustrate through numerical studies that our proposed method is more effective in detecting SNPs associated with a trait than either single-marker analysis using family data or model selection methods that ignore the familial dependency structure. Further, we perform gene-level analysis in Minnesota Center for Twin and Family Research (MCTFR) dataset using our method to detect several SNPs using this that have been implicated to be associated with alcohol consumption.

## Introduction

Genome Wide Association Studies (GWAS) have identified a large number of genetic variants associated with complex diseases^1,2^. The advent of economical high-throughput genotyping technology enables researchers to scan the genome with millions of Single Nucleotide Polymorphism (SNP)-s, and improvements in computational efficiency in analysis techniques has facilitated parsing through this huge amount of data to detect significant associations^3^. However, detecting small effects of individual SNPs requires large sample size^4^. For quantitative behavioral traits such as alcohol consumption, drug abuse, anorexia and depression, variation in genetic effects due to environmental heterogeneity brings in additional noise, further amplifying the issue. This is one of the motivations of performing GWAS on families instead of unrelated individuals, through which the environmental variation can be reduced^5–7^. However, association analysis of multiple SNPs while using dependent data with a familial structure and large sample sizes can be computationally very challenging. Thus single SNP association analysis is the standard tool for detecting SNPs, and most family studies tend to have smaller sample size. The MCTFR Study^6^ with genome-wide data on identical twins, non-identical twins, biological offspring, adoptees serve as the motivation for our methodology development in this paper.

A downside of family-based single-SNP methods—such as GRAMMAR^8^ and the association test of Chen and Abecasis^9^—is that they do not take into account shared environment effects *within* families. They assume that phenotypic similarity among individuals in a family is entirely due to their genetic similarity and not due to the effect of shared environment. As a result, they tend to lose power when analyzing data where shared environmental effects explain a substantial proportion of the total phenotypic variation (see^10,11^ for examples). The RFGLS method proposed by Li et al.^12^ does take into account genetic and environmental sources of familial similarity and provides fast inference through a rapid approximation of SNP-specific coefficients from a mixed effect model. However it is only able to handle single SNPs at a time.

Single-SNP methods are less effective in detecting SNPs with weak signals^4^. This is limiting in situations where multiple SNPs are jointly associated with the phenotype^13–15^. Several methods of multi-SNP analysis have been proposed as alternatives. The kernel based association tests^15–18^ are prominent among such techniques. However, all such methods test for whether a group of SNPs is associated with the phenotype of interest *as a whole*, and do not prioritize within that group to detect the individual SNPs primarily associated with the trait. One way to solve this problem is to perform model selection. The methods of Frommelet et al.^19^ and Zhang et al.^20^ take this approach, and perform SNP selection from a multi-SNP model on GWAS data from *unrelated individuals*. However, they rely on fitting models corresponding to multiple predictor sets, hence are computationally very intensive to implement in a linear mixed effect framework for modeling familial data.

In this paper we propose a fast and scalable model selection technique that fits a single model to a familial dataset, and aims to identify genetic variants with weak signals that are associated with the outcome through joint modelling of multiple variants. We consider only main effects of the variants, but this can be extended to include higher-order interactions. We achieve this by extending the framework of e-values^21^, which we discuss in “Feature selection with e-values”. There we present the definition of an e-value, discuss some of its properties, and describe how we generalize the e-values for our scenario. Broadly, for any estimation method that provides consistent estimates (at a certain rate relative to the sample size) of the vector of parameters, e-values quantify the proximity of the sampling distribution for a restricted parameter estimate to that of the full model estimate in a regression-like setup. A variable selection algorithm using the e-values has the three simple and generic steps. First, fit the full model, i.e. where all predictor effects are being estimated from the data, and use resampling to estimate its e-value. Second, set an element of the full model coefficient estimate to 0 and get an e-value for that predictor using resampling distribution of previously estimated parameters- repeat this for all predictors. Then finally, select predictors that have e-values below a pre-determined threshold.

The above algorithm offers multiple important benefits in the SNP selection scenario. Unlike other model selection methods, only the full model needs to be computed here. It thus offers the user more flexibility in utilizing a suitable method of estimation for the full model. Our method allows for fitting multi-SNP models, thereby accommodating cases of modelling multiple correlated SNPs or closely located multiple causal SNPs simultaneously. Finally, we use the Generalized Bootstrap (GBS)^22^ as our chosen resampling technique. Instead of fitting a separate model for each bootstrap sample, GBS computes bootstrap estimates using Monte-Carlo samples from the resampling distribution as weights, and reusing model objects obtained from the full model. Consequently, the resampling step becomes very fast and parallelizable.

In past literature, Vanderweele and Ding^23^ and Vovk and Wang^24^ used the term ‘e-value’ in the contexts of sensitivity analysis and multiple testing, respectively. In comparison, the e-values we use^21^ *evaluate* the relevance of a variable with reference to a statistical model. Going beyond the existing proposal of e-values tied to specific objectives and models, as well as the well-known *p*-values used for hypothesis testing, this e-value is assumption-lean, covers more generic statistical problems—such as including dependent data models—and is expandable to numerous applications, including group feature selection, hypothesis testing, and multiple testing.

## Materials and methods

### The MCTFR data

The familial GWAS dataset collected and studied by Minnesota Center for Twin and Family Research (MCTFR)^6,10,12^ consists of samples from three longitudinal studies conducted by the MCTFR: (1) the Minnesota Twin Family Study (MTFS)^25^ that covers twins and their parents, (2) the Sibling Interaction and Behavior Study (SIBS)^26^ that includes adopted and biological sibling pairs and their rearing parents, and (3) the enrichment study^27^ that extended the MTFS by oversampling 11 year old twins who are highly likely to develop substance abuse. While 9827 individuals completed the phenotypic assessments for participation in the study, after several steps of screening^6^ the genotype data from 7605 Caucasian individuals clustered in 2151 nuclear families were included in our analysis. This consisted of 1109 families where the children are identical twins, 577 families with non-identical twins, 210 families with two adopted children, 162 families with two non-twin siblings, and 93 families where one child is adopted while the other is the biological child of the parents.

DNA samples collected from the subjects were analyzed using Illumina’s Human660W-Quad Array, and after standard quality control steps^6^, 527,829 SNPs were retained. Covariates for each sample included age, sex, birth year, generation (parent or offspring), as well as the two-way interactions generation x age, generation x sex, and generation x birth year. Five quantitative phenotypes measuring substance use disorders were studied in this GWAS: (1) Nicotine dependence, (2) Alcohol consumption, (3) Alcohol dependence, (4) Illegal drug usage, and (5) Behavioral disinhibition. The response variables corresponding to these phenotypes are derived from questionnaires using a hierarchical approach based on factor analysis^28^.

A detailed description of the data is available in Miller et al.^6^. Several studies reported SNPs associated with phenotypes collected in MCTFR study^10,12,29^. Li et al.^12^ used RFGLS to detect association between height and genetic variants through single-SNP analysis, while^10^ used the same method to study SNPs influencing the development of all five indicators of behavioral disinhibition mentioned above. Irons^30^ focused on the effect of several factors affecting alcohol use in the study population, namely the effects of polymorphisms in the ALDH2 gene and the GABA system genes, as well as the effect of early exposure to alcohols as adolescents to adult outcomes. Finally Coombes et al.^29^ used a bootstrap-based combination test and a sequential score test to evaluate gene-environment interactions for alcohol consumption.

### Consents and approvals

Data were collected through the Minnesota Center for Twin and Family Research (MCTFR). All University of Minnesota and National Institute of Health (NIH) guidelines for human subjects research were followed in the collection and processing of the data. The protocol was approved by the Institutional Review Board (IRB) at the University of Minnesota (protocol # 0303M45703). Participants aged 18 years and older completed informed consent, while consent was obtained from at least one parent for those participants younger than 18 and the minor participant also assented to participate.

### Statistical model

We use a Linear Mixed Model (LMM) with three variance components accounting for several potential sources of variation to model effect of SNPs behind a quantitative phenotype. This is known as *ACE model* in the literature^31^. While the-state-of-the-art focuses on detection of a *single variant at a time*, we will incorporate *all* SNPs genotyped within a gene (or group of genes in some cases) as set of fixed effects in a *single model*.

Following standard protocol for family-based GWAS^9,10,12^, we assume a data setting of nuclear pedigrees, i.e. that the data consists of observations from individuals of multiple genetically unrelated families, with individuals within a family potentially sharing genetic material. Suppose there are *m* such families in total, with the $i^{\mathop {\textrm{th}}\limits }$ pedigree containing $n_i$ individuals , and the total number of individuals is $n = \sum _{i=1}^m n_i$. Denote by ${y}_i = (y_{i 1}, \ldots , y_{i n_i})^T$ the quantitative trait values for individuals in that pedigree, while the matrix ${\textbf{G}}_i \in \mathbb {R}^{ n_i \times p_g}$ contains their genotypes for a number of SNPs. Let ${\textbf{C}}_i \in \mathbb {R}^{ n_i \times p}$ denote the data on *p* covariates for individuals in the pedigree *i*. Given these, we consider the following model.

1 $$\begin{aligned} {\textbf{Y}}_i = \alpha + {\textbf{G}}_i {\beta }_g + {\textbf{C}}_i {\beta }_c + {\epsilon }_i, \end{aligned}$$

with $\alpha$ the intercept term, ${\beta }_g$ and ${\beta }_c$ fixed coefficient terms corresponding to the multiple SNPs and covariates, respectively, and ${\epsilon }_i \sim \mathcal {N}_{n_i} (\textbf{0}, {\textbf{V}}_i)$ the random error term. To account for the within-family dependency structure, we break up the random error variance into three independent components:

2 $$\begin{aligned} {\textbf{V}}_i = \sigma _a^2 {\varvec{\Phi }}_i + \sigma _c^2 \textbf{1} \textbf{1}^T + \sigma _e^2 {\textbf{I}}_{n_i}. \end{aligned}$$

The three components of ${\textbf{V}}_i$ in (2) model different sources of random variations that can affect the quantitative trait values for individuals in the $i^{\mathop {\textrm{th}}\limits }$ pedigree. The first component above is a within-family random effect term to account for shared polygenic effects. The proportion of of genetic material shared between pairs of individuals in a family is represented by elements of the matrix ${\varvec{\Phi }}_i$. Its $(s,t)^{\mathop {\textrm{th}}\limits }$ element represents two times the kinship coefficient, which is the probability that two alleles, one randomly chosen from individual *s* in pedigree *i* and the other from individual *t*, are ‘identical by descent’, i.e. come from same common ancestor^31^. Following basic probability, the kinship coefficient of a parent-child pair is 1/4, a full sibling pair or non-identical (or dizygous = DZ) twins is 1/4, and for identical (or monozygous = MZ) twins is 1/2 in a nuclear pedigree. Following this, we can construct the ${\varvec{\Phi }}_i$ matrices for different types of families:

$$\begin{aligned} {\varvec{\Phi }}_{MZ} = \begin{bmatrix} 1 &{} 0 &{} 1/2 &{} 1/2 \\ 0 &{} 1 &{} 1/2 &{} 1/2 \\ 1/2 &{} 1/2 &{} 1 &{} 1\\ 1/2 &{} 1/2 &{} 1 &{} 1 \end{bmatrix}, {\varvec{\Phi }}_{DZ} = \begin{bmatrix} 1 &{} 0 &{} 1/2 &{} 1/2 \\ 0 &{} 1 &{} 1/2 &{} 1/2 \\ 1/2 &{} 1/2 &{} 1 &{} 1/2\\ 1/2 &{} 1/2 &{} 1/2 &{} 1 \end{bmatrix}, {\varvec{\Phi }}_{Adopted} = {\textbf{I}}_4. \end{aligned}$$

for families with parents (indices 1 and 2) and MZ twins, DZ twins, or two adopted children (indices 3 and 4), respectively.

The second variance component $\sigma _c^2 \textbf{1} \textbf{1}^T$ in (2) accounts for shared environmental effect within each pedigree. Traits of each individual in the pedigree are affected by the same amount—a single random draw from $N(0,\sigma _c^2)$—of random variation. The third term in (2) quantifies other sources of variation unique to each individual.

### Feature selection with e-values

We extend the recently-proposed framework of e-values^21^ to select important SNPs in the above gene-level, multi-SNP statistical model. In a general modelling situation where one needs to estimate a set of parameters ${\theta }\in \mathbb {R}^d$ from data with sample size *n*, a statistical model corresponds to a subset of the full parameter space. In other words, the estimable index set of $\theta$, say $\mathcal {S}\subseteq \{ 1, \ldots , d \}$ specifies a model. The other indices are set at constant values—typically in model selection literature the constants are set at 0. Note that we are attempting to select important SNPs as described in “Statistical model”, thus in our setting $\theta \equiv \beta _g, d \equiv p_g$.

Following the recipe in Majumdar and Chatterjee^21^, we obtain coefficient estimates corresponding to model $\mathcal {S}$ by simply replacing elements of the ‘full model’ estimate ${\hat{\theta }}$—i.e. the the coefficient estimate with all possible parameters included—at indices not in $\mathcal {S}$:

$$\begin{aligned} {\hat{\theta }}_{\mathcal {S}} = \left\{ \begin{array}{ll} {\hat{\theta }}_{j} &{} \text { for } j \in \mathcal {S}, \\ 0 &{} \text { for } j \notin \mathcal {S}. \end{array} \right. \end{aligned}$$

*Sampling distribution* is defined as the distribution of a parameter estimate, based on the random data samples used to calculate this estimate. We compare sampling distributions of the above model with the full model, i.e. $[{\hat{{\theta }}}_\mathcal {S}]$ with $[{\hat{{\theta }}}]$ (denoting the distribution of a random variable by $[\cdot ]$). For this comparison, we define an *evaluation map* function $E: \mathbb {R}^d \times {\tilde{\mathbb {R}}}^d \rightarrow [0, \infty )$ that measures the relative position of ${\hat{{\theta }}}_\mathcal {S}$ with respect to $[{\hat{{\theta }}}]$. Here ${\tilde{\mathbb {R}}}^d$ is the set of probability measures on $\mathbb {R}^d$. For any ${x}\in \mathbb {R}^d$ and $[ {\textbf{X}}] \in {\tilde{\mathbb {R}}}^d$ with a positive definite covariance matrix $\mathbb {V}{\textbf{X}}$, we consider the following evaluations functions in this paper:

3 $$\begin{aligned} E_1 ({x}, [ {\textbf{X}}])&= \left[ 1 + \left\| ({\textrm{diag}}(\mathbb {V}{\textbf{X}}))^{-1/2} \odot ({x}- \mathbb {E}{\textbf{X}}) \right\| ^2 \right] ^{-1}, \end{aligned}$$

4 $$\begin{aligned} E_2 ({x}, [ {\textbf{X}}])&= \exp \left[ - \left\| ({\textrm{diag}}(\mathbb {V}{\textbf{X}}))^{-1/2} \odot ({x}- \mathbb {E}{\textbf{X}}) \right\| \right] . \end{aligned}$$

Here ${\textrm{diag}}(\mathbb {V}{\textbf{X}})$ denotes the vector composed of the diagonal entries in $\mathbb {V}{\textbf{X}}$, and $\odot$ represents elementwise product, so that $({\textrm{diag}}(\mathbb {V}{\textbf{X}}))^{1/2}$ is the vector of coordinate-wise standard deviation, and $({\textrm{diag}}(\mathbb {V}{\textbf{X}}))^{-1/2} \odot ({x}- \mathbb {E}{\textbf{X}})$ is a normalized version of ${x}$. Data depths^32,33^ also constitute a broad class of functions that can be used as evaluation maps—as done by Majumdar and Chatterjee^21^. In general, any continuous function that is location and scale invariant, and has a few basic convergence properties is a good choice for the evaluation map function (see conditions E1–E4 in the supplementary material).

#### Formulation

Note that the evaluation map function is defined conditional on a fixed value of ${\hat{\theta }}_\mathcal {S}$. Since ${\hat{\theta }}_\mathcal {S}$ itself has a distribution, so does the evaluation map. We define the **e-value** as any functional of the evaluation map distribution $\mathbb {E}_{\mathcal {S}} \equiv [ E( {\hat{\theta }}_\mathcal {S}, [{\hat{\theta }}]) ]$ that can act as a measure of comparison between the sampling distributions of ${\hat{\theta }}_\mathcal {S}$ and ${\hat{\theta }}$. For example, Majumdar and Chatterjee^21^ took the mean functional of $\mathbb {E}_{\mathcal {S}}$ (say $\mu (\mathbb {E}_{\mathcal {S}})$) as e-value, and showed that it can be used as a model selection criterion. To this end, non-zero indices (say $\mathcal {S}_0$) of the true parameter vector ${\theta }_0$ can be recovered through a fast algorithm that has these generic steps:
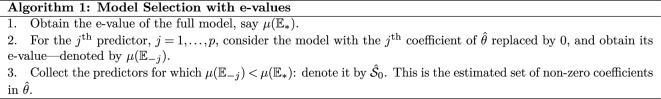


As $n \rightarrow \infty$, the above algorithm provides consistent model selection, i.e. $\mathbb {P}( {\hat{\mathcal {S}}}_0 = \mathcal {S}_0) \rightarrow 1$. In practice we only have one dataset, so it is not possible to access the true sampling distribution of ${\hat{\theta }}$ and ${\hat{\theta }}_\mathcal {S}$ to do the above. To this end, we use a fast bootstrap algorithm, called Generalized Bootstrap (GBS)^22^, to obtain approximations of the sampling distributions $[{\hat{\theta }}_\mathcal {S}], [{\hat{\theta }}]$, the evaluation map distributions, and the e-values. GBS is dependent of a tuning parameter $\tau _n$ that represents the standard deviation of the synthetic noise introduced by the bootstrap procedure. Intermediate values of $\tau _n$, such that $\tau _n/n \rightarrow \infty$, result in model selection consistency as described above.

#### Quantile e-values

When true signals are weak, the above method of variable selection leads to very conservative estimates of non-zero coefficient indices, i.e. a large number of false positives in a sample setting. This happens because the true values leave-one-covariate-out e-values for variables that correspond to small but non-zero coefficients in $\theta _0$ (hence weak signal) fall too close to the full model e-value. Consequently, when these e-values are estimated from randomly sampled data, simply by random chance their values can be slightly less than the full model e-value estimate.

**Figure 1:**
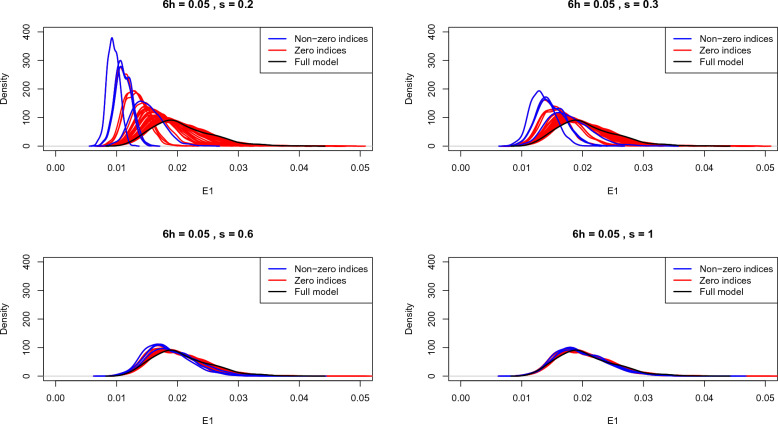
Density plots of bootstrap approximations for $\mathbb {E}_{* }$ and $\mathbb {E}_{-j}$ for all *j* in simulation setup, with $s = 0.2, 0.3, 0.6, 1$.

Figure 1 demonstrates this phenomenon in our setup, where we would like to estimate non-zero elements of the fixed effect coefficient vector $\beta _g$ in the model (1), i.e. $\beta _g \equiv \theta$. Here we analyze data on 250 families with monozygotic twins, each individual being genotyped for 50 SNPs. Four of these 50 SNPs are causal: each having a heritability of $h/6\%$ with respect to the total error variation present. The four panels show density plots of $\mathbb {E}_{-j}$ for $j = 1, \ldots , p$, as well as $\mathbb {E}_{* }$: estimated based on resampling schemes with four different values of the standard deviation parameter $s \equiv s_n = \tau _n / \sqrt{n}$. Focusing on where the central regions of the evaluation map distributions are, we notice that for smaller values of *s* there is quite a bit of overlap along the bootstrap estimates of $\mathbb {E}_{-j}$ for causal vs. non-causal SNPs. On the other hand, for large values of *s* all the density plots become essentially the same as the full model.

However, notice that the evaluation map distributions for non-zero vs. zero indices have different tail behaviors at smaller values of *s*. In the supplementary material S1 we show that the means and tail quantiles for $\mathbb {E}_{-j}$ and $\mathbb {E}_{*}$ asymptotically converge to different limits (Theorems A.1 and A.2), and these limits are well-approximated with a GBS scheme having small standard deviation *s* (Theorem A.3). A potential reason for the different tail behaviors we see above is that the convergence at tail quantiles happens at a faster rate than convergence at the means.

Consequently, instead of comparing means of the distributions, comparing a suitable tail quantile across the distributions is more likely to provide a better separation of non-zero vs. zero indices. For this reason we use tail quantiles as e-values.

When the $q^\text {th}$ quantile (denoted by $c_q$, $q \in (0,1)$) is taken as the e-value instead of the mean, we set a lower detection threshold than the same functional on the full model, i.e. choose all *j* such that

5 $$\begin{aligned} c_q (\mathbb {E}_{-j})< t c_q (\mathbb {E}_{*}), \quad 0< t < 1, \end{aligned}$$

to be included in the model. The optimal choice of *q* and *t* depends on factors such as specifications of the statistical model, sample size, and degree of sparsity of parameters in the data generating process. We demonstrate this point through our experiments in “Experiments”. For *q*, we take the conservative route by only flagging a SNP as ‘detected’ if Eq. (5) holds for *all* $q \in \{0.5, 0.6, 0.7, 0.8, 0.9\}$. This approach leads to a tradeoff between the true positive and true negative SNP detections rates for different values of *t*. We demonstrate this fact through synthetic data experiments (“Synthetic data”), and choose the best *t* that minimizes prediction error on a holdout sample in the MCTFR data analysis (“Analysis of the MCTFR data”).

## Experiments

We now evaluate the performance of the above formulation of quantile e-values in through on synthetic data, as well as the MCTFR Twin Studies dataset.

### Synthetic data

Consider the model in (1) with no environmental covariates and familes with MZ twins. We take a total of $p_g = 50$ SNPs, and generate the SNP matrices ${\textbf{G}}_i$ in correlated blocks of 6, 4 ,6, 4 and 30 to simulate correlation among SNPs in the genome. We set the correlation between two SNPs inside a block at 0.7, and consider the blocks to be uncorrelated. For each parent we generate two independent vectors of length 50 with the above correlation structure, and entries within each block being 0 or 1 following Bernoulli distributions with probabilities 0.2, 0.4, 0.4, 0.25 and 0.25 (Minor Allele Frequency or MAF) for SNPs in the 5 blocks, respectively. The genotype of a person is then determined by taking the sum of these two vectors: thus entries in ${\textbf{G}}_i$ can take the values 0, 1 or 2. Finally we set the common genotype of the twins by randomly choosing one allele vector from each of the parents and taking their sum.

We repeat the above process for $m=250$ families. In GWAS generally each associated SNP explains only a small proportion of the overall variability of the trait. To reflect this in our simulation setup, we assume that the first entries in each of the first four blocks above are causal, and each of them explains $h/(\sigma _a^2+\sigma _c^2+\sigma _e^2) \%$ of the overall variability. The term *h* is known as the *heritability* of the corresponding SNP. The value of the non-zero coefficient in *k*-th block: $k = 1,\ldots , 4$, say $\beta _k$ is calculated using the formula:

6 $$\begin{aligned} \beta _k = \sqrt{ \frac{h}{100 (\sigma _a^2+\sigma _c^2+\sigma _e^2). 2 \text {MAF}_k (1 - \text {MAF}_k) }}. \end{aligned}$$

We fix the following values for the error variance components: $\sigma _a^2 = 4, \sigma _c^2 = 1, \sigma _e^2 = 1$, and generate pedigree-wise response vectors ${y}_1, \ldots , {y}_{250}$ using the above setup. To consider different SNP effect sizes, we repeat the above setup for $h \in \{10, 7, 5, 3, 2, 1, 0 \}$, generating 1000 datasets for each value of *h*.

**Competing methods** We compare our e-value based approach using the evaluation maps $E_1$ and $E_2$ in (3) with two groups of methods:

*(1) Model selection on linear model:* Here we ignore the dependency structure within families by training linear models on the simulated data and selecting SNPs with non-zero effects by backward deletion using a modification of the BIC called mBIC2. This has been showed to give better results than single-SNP analysis in a GWAS with unrelated individuals^19^ and provides approximate False Discovery Rate (FDR) control^34^.

*(2) Single-marker mixed model:* We train single-SNP versions of (1) using a fast approximation of the Generalized Least Squares procedure (named Rapid Feasible Generalized Least Squares or RFGLS^12^), obtain marginal *p*-values from corresponding *t*-tests and use two methods to select significant SNPs: the Benjamini-Hochberg (BH) procedure, as well as the Local FDR method^35^ (LFDR).

For mBIC2 and BH, we choose a conservative FDR level of 0.05 guided by choices in existing work^36,37^. Higher FDR values lead to marginal increase in true positive rate but sharp decreases in true negative rate (see definitions of these metrics below). LFDR tends to be much more conservative than global FDR procedures, so we repeated its simulations for a range of FDR values in $\{0.01, 0.05, 0.1, 0.2, 0.3, 0.4, 0.5\}$. Table 1 shows the performance metrics at FDR = 0.4, the highest value where the true negative rate is at least as much as the lowest true negative across all settings of e-values as described below.

With the e-value being the $q^\text {th}$ quantile of the evaluation map distribution, we set the detection threshold value at the $t^\text {th}$ multiple of *q* for some $0< t < 1$. This means all indices *j* such that the $q^\text {th}$ quantile of the bootstrap approximation of $\mathbb {E}_{-j}$ is less than the $tq^\text {th}$ quantile of the bootstrap approximation of $\mathbb {E}_{*}$ get selected as the set of active predictors. To enforce stricter control on the selected set of SNPs we repeat this for $q \in \{ 0.5, 0.6, 0.7, 0.8, 0.9 \}$, and take the SNPs that get selected for *all* values of *q* as the final set of selected SNPs. Guided by empirical experiments, we chose values of *t* for $E_1$ and $E_2$ that clearly demonstrate the tradeoff of TP/TN (or RTP/RTN) rates for the e-values.

Since the above procedure depends on the bootstrap standard deviation parameter *s*, we repeat the process for $s \in \{ 0.3, 0.15, \ldots , 0.95, 2 \}$, and take as the final estimated set of SNPs the SNP set ${\hat{\mathcal {S}}}_t (s)$ that minimizes fixed effect prediction error (PE) on an independently generated test dataset $\{ ({y}_{test,i}, {\textbf{G}}_{test,i}), i = 1, \ldots , 250 \}$ from the same setup above:

$$\begin{aligned} \text {PE}_t (s)&= \sum _{i=1}^{250} \sum _{j=1}^4 \left( y_{test,ij} - {g}_{test,ij}^T {\hat{{\beta }}}_{{\hat{\mathcal {S}}}_t (s)} \right) ^2; \\ {\hat{\mathcal {S}}}_t&= \mathop {\mathrm {arg\,min}}\limits _s \text {PE}_t (s) \end{aligned}$$

**Metrics** We use the following metrics to evaluate each method we implement: (1) True Positive (TP), which is the proportion of causal SNPs detected; (2) True Negative (TN), which is the proportion of non-causal SNPs undetected; (3) Relaxed True Positive (RTP), which is the: proportion of detecting any SNP in each of the 4 blocks with causal SNPs, i.e. for the selected index set by some method *m*, say ${\hat{\mathcal {S}}}_m$,

$$\begin{aligned} \text {RTP} ( {\hat{\mathcal {S}}}_m ) = \frac{1}{4} \sum _{i=1}^4 \mathbb {I}( \text {Block } i \cap {\hat{\mathcal {S}}}_m \ne \emptyset ), \end{aligned}$$

and finally (4) Relaxed True Negative (RTN), which is the proportion of SNPs in block 5 undetected. We consider the third and fourth metrics to cover situations in which the causal SNP is not detected itself, but highly correlated SNPs with the causal SNP are. This is common in GWAS^19^. We average the above proportions over 1000 replications, and repeat the process for two different ranges of *t* for $E_1$ and $E_2$.

**Results** We present the simulation results in Table 1. For all heritability values, applying mBIC2 on linear models performs poorly compared to applying RFGLS and then correcting for multiple testing. This is expected because the linear model ignores within-family error components.

**Table 1:** (Top) Average true positive (TP)/l(TN) rates for mBIC2, RFGLS+BH and the e-values method with $E_1$ and $E_2$ as evaluation maps and different values of *t* over 1000 replications, and (Bottom) average relaxed true positive (RTP) and relaxed true negative (RTN) rates.

Method		$h = 10$	$h = 7$	$h = 5$	$h = 3$	$h = 2$	$h = 1$	$h = 0$
mBIC2		0.79/0.99	0.59/0.99	0.41/0.99	0.2/0.99	0.11/0.99	0.05/0.99	-/0.99
RFGLS+BH		0.95/0.92	0.82/0.95	0.62/0.97	0.29/0.98	0.14/0.99	0.04/1	-/1
RFGLS+LFDR		0.54/0.99	0.46/0.99	0.39/0.99	0.29/0.99	0.23/1	0.15/1	-/0.96
$E_1$	$t = \exp (-1)$	0.95/0.98	0.87/0.97	0.74/0.97	0.47/0.97	0.28/0.97	0.12/0.98	-/0.99
	$t = \exp (-2)$	0.94/0.98	0.85/0.98	0.69/0.98	0.43/0.98	0.25/0.98	0.09/0.99	-/0.99
	$t = \exp (-3)$	0.94/0.99	0.82/0.98	0.65/0.98	0.37/0.99	0.2/0.99	0.07/0.99	-/1
	$t = \exp (-4)$	0.92/0.99	0.79/0.99	0.61/0.99	0.32/0.99	0.17/0.99	0.06/1	-/1
	$t = \exp (-5)$	0.9/0.99	0.75/0.99	0.55/0.99	0.26/1	0.13/1	0.04/1	-/1
$E_2$	$t = 0.8$	0.97/0.98	0.9/0.97	0.79/0.96	0.54/0.96	0.34/0.97	0.15/0.98	-/0.99
	$t = 0.74$	0.96/0.98	0.88/0.97	0.75/0.97	0.48/0.97	0.29/0.98	0.12/0.98	-/0.99
	$t = 0.68$	0.95/0.99	0.87/0.98	0.72/0.98	0.45/0.98	0.26/0.98	0.1/0.99	-/0.99
	$t = 0.62$	0.95/0.99	0.84/0.98	0.68/0.98	0.4/0.99	0.22/0.99	0.09/0.99	-/0.99
	$t = 0.56$	0.94/0.99	0.82/0.99	0.65/0.99	0.36/0.99	0.19/0.99	0.07/1	-/1
	$t = 0.5$	0.92/0.99	0.79/0.99	0.6/0.99	0.31/0.99	0.16/1	0.05/1	-/1

Method		$h = 10$	$h = 7$	$h = 5$	$h = 3$	$h = 2$	$h = 1$	$h = 0$
mBIC2		0.84/0.99	0.66/0.99	0.48/0.99	0.26/0.99	0.16/0.99	0.08/0.99	–/0.98
RFGLS+BH		0.96/0.99	0.83/0.99	0.64/0.99	0.32/0.99	0.16/1	0.05/1	–/1
RFGLS+LFDR		0.55/0.99	0.47/0.99	0.42/0.99	0.37/0.99	0.35/1	0.31/1	–/0.97
$E_1$	$t = \exp (-1)$	0.95/0.98	0.87/0.97	0.75/0.97	0.5/0.97	0.32/0.98	0.15/0.98	–/0.98
	$t = \exp (-2)$	0.94/0.99	0.85/0.98	0.71/0.98	0.45/0.98	0.28/0.98	0.12/0.99	–/0.98
	$t = \exp (-3)$	0.94/0.99	0.83/0.99	0.67/0.99	0.39/0.99	0.22/0.99	0.09/0.99	–/0.99
	$t = \exp (-4)$	0.92/0.99	0.8/0.99	0.62/0.99	0.33/0.99	0.18/0.99	0.07/1	–/1
	$t = \exp (-5)$	0.9/0.99	0.75/0.99	0.56/0.99	0.27/1	0.14/1	0.05/1	–/1
$E_2$	$t = 0.8$	0.97/0.98	0.91/0.97	0.8/0.96	0.57/0.96	0.38/0.97	0.2/0.98	–/0.97
	$t = 0.74$	0.96/0.98	0.89/0.98	0.76/0.97	0.51/0.97	0.33/0.98	0.15/0.98	–/0.98
	$t = 0.68$	0.95/0.99	0.87/0.98	0.73/0.98	0.48/0.98	0.29/0.98	0.12/0.99	–/0.98
	$t = 0.62$	0.95/0.99	0.85/0.99	0.69/0.98	0.42/0.99	0.24/0.99	0.11/0.99	–/0.99
	$t = 0.56$	0.94/0.99	0.83/0.99	0.66/0.99	0.38/0.99	0.2/0.99	0.08/0.99	–/0.99
	$t = 0.5$	0.92/0.99	0.79/0.99	0.61/0.99	0.32/0.99	0.17/1	0.06/1	–/1

Our method works better than the two competing methods for detecting true signals across different values of *h*: the average TP rate going down slowly than other methods across the majority of choices for *t*. All the competing methods (mBIC2, RFGLS+BH, RFGLS+LFDR) have very high true negative detection rates, which is matched by our method for higher values of *q*. Since all reduced model distributions reside on the left of the full model distribution, we expect the variable selection process to turn more conservative at lower values of *t*. This effect is more noticeable for lower *q*, indicating that the right tails of evaluation map distributions are more useful for this purpose. Finally for $h=0$, we report only TN and RTN values since no signals should ideally be detected. Note also the fact that we report the performance metrics for $E_1, E_2$ considering a very conservative selection process: we only mark a SNP *j* as ‘detected’ if $c_q (\mathbb {E}_{-j}) < t c_q (\mathbb {E}_{*})$ for *all* $q \in \{ 0.5, 0.6, 0.7, 0.8, 0.9 \}$. We experimentally observed that relaxing this condition leads to higher strict TP rates but lower strict TN.

RTP performances for all methods are better than the corresponding TP/TN performances. However, for mBIC2 this seems to be due to detecting SNPs in the first four blocks by chance since for $h=0$ its RTN is less than TN. Also $E_2$ seems to perform slightly better than $E_1$, in the sense that it yields a higher TP (or RTP) while having the same TN (or RTN) rates.

Among competing methods, it is interesting to notice that LFDR performs better than the other two for small signal values ($h \le 3$), but worse at higher *h*. This speaks to the strengths of LFDR in low-signal situations. On the other hand, LFDR is calculated using density estimates of the null and non-null statistic distributions. Since there are only 50 SNPs in our simulation setting (and even less in the real data setting), the resulting instability is a potential reason for its low performance at high values of *h*.

### Analysis of the MCTFR data

We now apply the above methods on SNPs from the MCTFR dataset. We assume a nuclear pedigree structure, and for simplicity only analyze pedigrees with MZ and DZ twins. After setting aside samples with missing response variables, we end up with 1019 such 4-member families. We look at the effect of genetic factors behind the response variable pertaining to the amount of alcohol consumption, which is highly heritable in this dataset according to previous studies^10^. We analyze SNPs inside some of the most-studied genes with respect to alcohol abuse: GABRA2, ADH1A, ADH1B, ADH1C, ADH4-ADH7, SLC6A3, SLC6A4, OPRM1, CYP2E1, DRD2, ALDH2, and COMT^38^ through separate gene-level models. None of the ADH genes contained a sufficient number of SNPs to justify analysis individually, so we pooled SNPs across all 7 ADH genes for analysis. We include sex, birth year, age and generation (parent or offspring) of individuals as covariates to control for their potential effect.

For model selection we use $E_2$ as the evaluation function because of its slighty better performance in the simulations. For each gene-level model, We train the LMM in (1) on 75% of randomly selected families, perform our e-values procedure for $s = 0.2, 0.4, \ldots , 2.8, 3, t = 0.1, 0.15, \ldots , 0.75, 0.8$; and select the set of SNPs that minimizes fixed effect prediction error on the data from the other 25% of families over this grid of (*s*, *t*). Note that we consider a wider range of *t* than in the simulations. This is because of the fact that instead of demonstrating the tradeoff of true positive and true negative rates, our objective here is to actually choose a set of SNPs. For the competing methods, we set FDR levels at 0.05 for mBIC2 and RFGLS+BH, while choose the level that minimizes fixed effect prediction error on the same holdout data as above for RFGLS+LFDR.

**Table 2:** Table of analyzed genes and number of detected SNPs in them by the three methods.

Gene	Total no. of SNPs	No. of SNPs detected by			
		e-value	RFGLS+BH	RFGLS+LFDR	mBIC2
GABRA2	11	5	0	1	0
ADH	44	3	1	1	0
OPRM1	47	25	1	0	0
CYP2E1	9	5	0	0	0
ALDH2	6	5	0	1	1
COMT	15	14	0	1	0
SLC6A3	18	4	0	1	0
SLC6A4	5	0	0	1	0
DRD2	17	0	0	0	1

As seen in Table 2, our e-value based technique detects a much higher number of SNPs than the two competing methods. Our method selects all but one SNP in the genes ALDH2 and COMT. These are small genes of size 50kb and 30kb, respectively, thus SNPs within them have more chance of being in high Linkage Disequilibrium (LD). On the other hand, it does not select any SNPs in SLC6A4 and DRD2. Variants of these genes are known to interact with each other and are jointly associated with multiple behavioral disorders^39,40^.

**Table 3:** Table of detected SNPs with known references.

Gene	Detected SNPs with known associations	Reference for associated SNP
GABRA2	rs1808851, rs279856: close to rs279858	Cui et al.^41^
ADH genes	rs17027523: 20kb upstream of rs1229984	Multiple studies (https://www.snpedia.com/index.php/Rs1229984)
OPRM1	rs12662873: 1 kb upstream of rs1799971	Multiple studies (https://www.snpedia.com/index.php/Rs1799971)
CYP2E1	rs9419624: 600b downstream of rs4646976; rs9419702: 10kb upstream of rs4838767	Lind et al.^42^
ALDH2	rs16941437: 10kb upstream of rs671	Multiple studies (https://www.snpedia.com/index.php/Rs671)
COMT	rs4680, rs165774	Voisey et al.^43^
SLC6A3	rs464049	Huang et al.^44^

A number of SNPs we detect (or SNPs situated close to them) have known associations with alcohol-related behavioral disorders. We summarize this in Table 3. Prominent among them are rs1808851 and rs279856 in the GABRA2 gene, which are at perfect LD with rs279858 in the larger, 7188-individual version of our twin studies dataset^30^. This SNP is the marker in GABRA2 that is most frequently associated in the literature with alcohol abuse^41^, but was not genotyped in our sample. A single SNP RFGLS analysis of the same twin studies data that used Bonferroni correction on marginal *p*-values missed the SNPs we detect^30^: highlighting the advantage of our approach. We give a gene-wise discussion of associated SNPs, as well as information on all SNPs, in the supplementary material S1.

**Figure 2:**
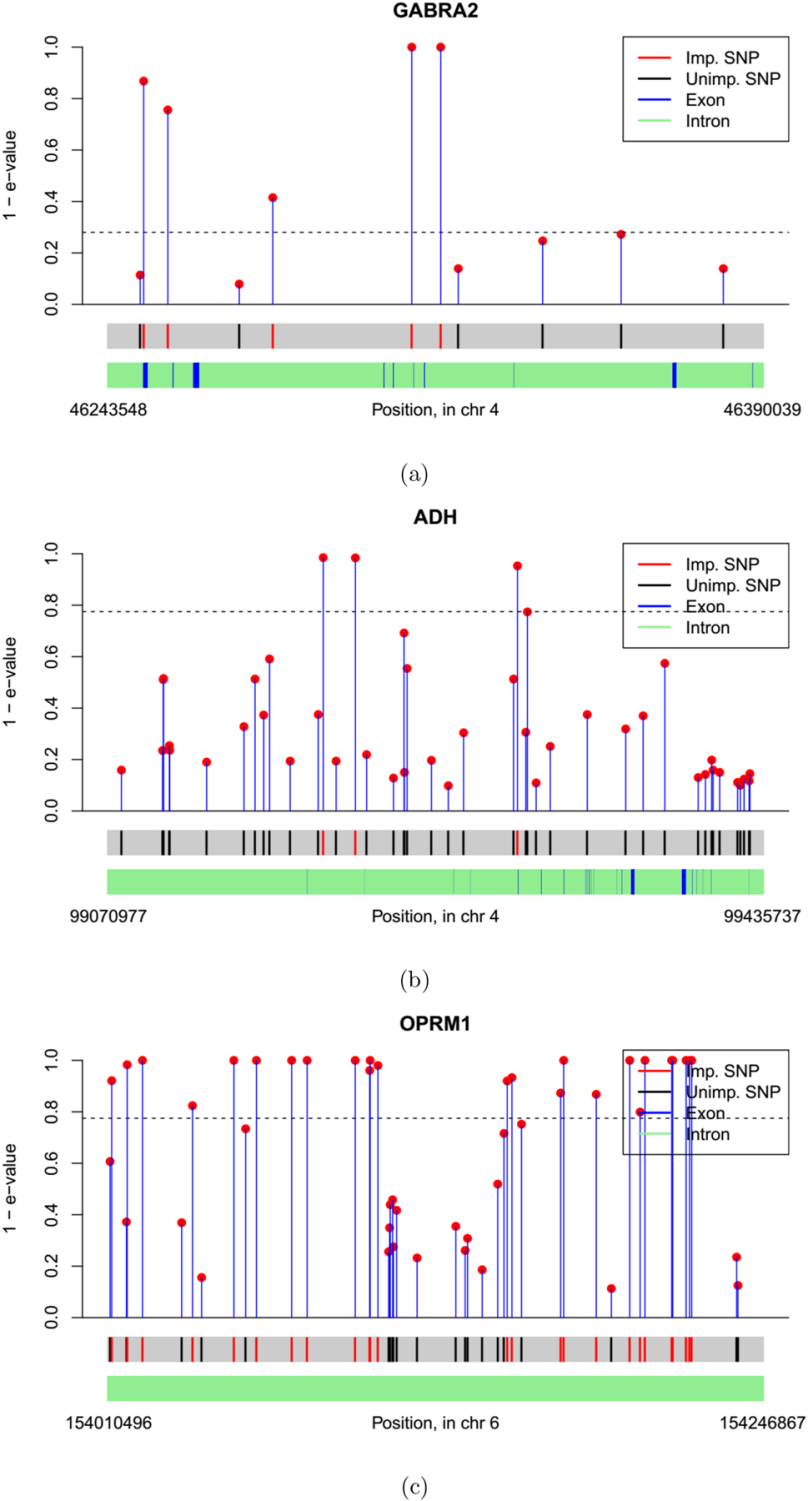
Plot of e-values for genes analyzed: (**a**) GABRA2, (**b**) ADH1–ADH7, (**c**) OPRM1. For ease of visualization, $1 - e$-values are plotted in the y-axis.

**Figure 3:**
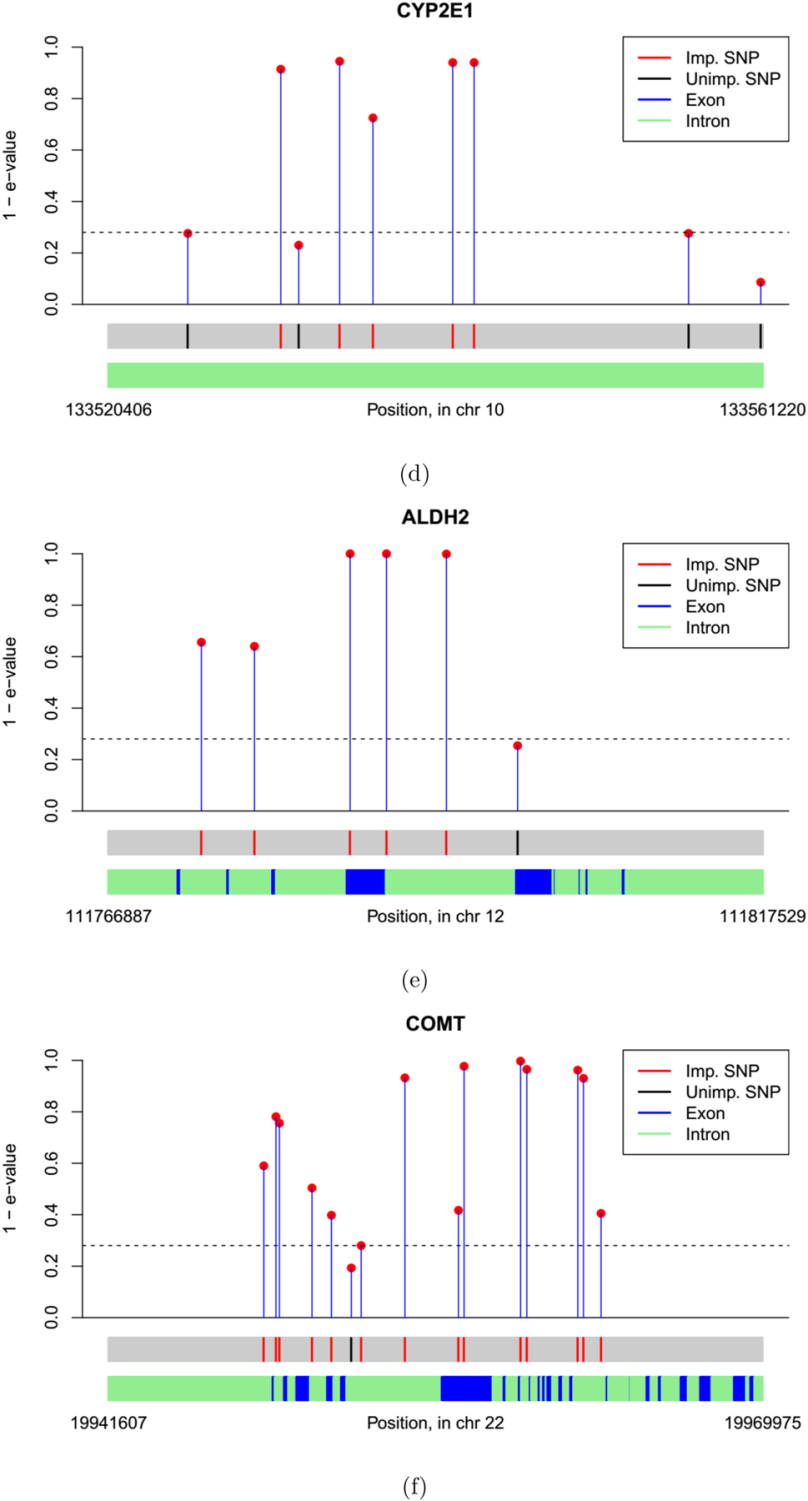
Plot of e-values for genes analyzed: (**d**) CYP2E1, (**e**) ALDH2, (**f**) COMT.

**Figure 4:**
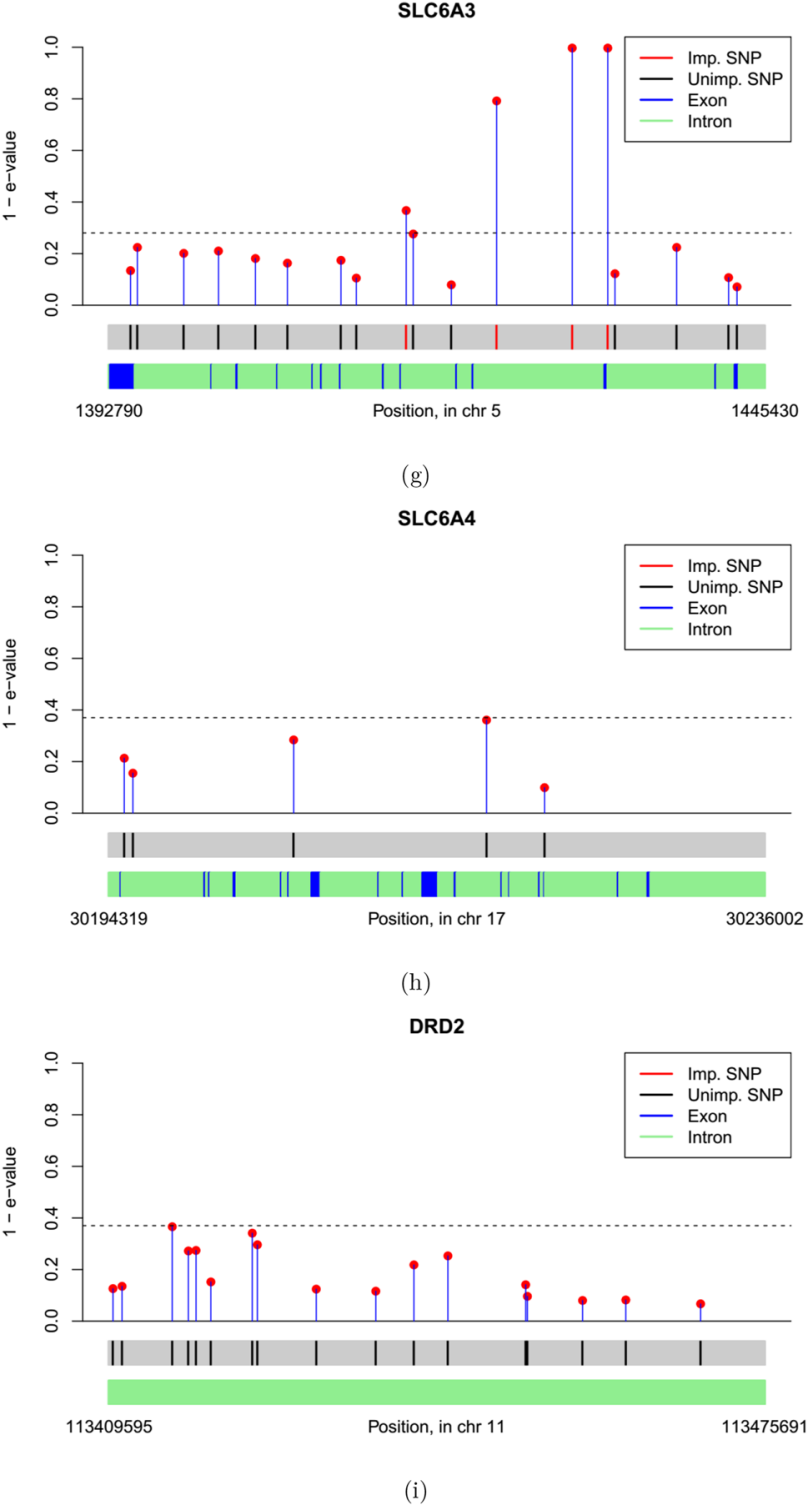
Plot of e-values for genes analyzed: (**g**) SLC6A3, (**h**) SLC6A4, (**i**) DRD2.

We plot the $90^{\mathop {\textrm{th}}\limits }$ quantile e-value estimates in Figs. 2, 3 and 4. We obtained gene locations, as well as the locations of coding regions of genes, i.e. exons, inside 6 of these 9 genes from annotation data extracted from the UCSC Genome Browser database^45^. Exon locations were not available for OPRM1, CYP2E1 and DRD2. In general, SNPs tend to get selected in groups with neighboring SNPs, which suggests high LD. Also most of the selected SNPs either overlap or in close proximity to the exons, which underline their functional relevance.

## Discussion and conclusion

To expand the above approach to a genome-wide scale, we need to incorporate strategies for dealing with the hierarchical structure of pathways and genes: there are only a few genes associated with a quantitative phenotype, which can be further attributed to a small proportion of SNPs inside each gene. To apply the e-values method here, it is plausible to start with an initial screening step to eliminate evidently non-relevant genes. Methods like the grouped Sure Independent Screening^46^ and min-P test^47^ can be useful here. Following this, in a multi-gene predictor set, there are several possible strategies to select important genes *and* important SNPs in them. Firstly, one can use a two-stage e-value based procedure. The first stage is same as the method described in this paper, i.e. selecting important SNPs from each gene using multi-SNP models trained on SNPs in that gene. In the second stage, a model will be trained using the aggregated set of SNPs obtained in the first step, and a group selection procedure will be run on this model using e-values. This means dropping *groups* of predictors (instead of single predictors) from the full model, checking the reduced model e-values, and selecting a SNP group only if dropping it causes the e-value to go below a certain cutoff. Secondly, one can start by selecting important genes using an aggregation method of SNP-trait associations (e.g. Lamparter et al.^48^) and then run the e-value based SNP selection on the set of SNPs within these genes. Thirdly, one can also take the aggregated set of SNPs obtained from running the e-values procedure on gene-level models, then use a fast screening method (e.g. RFGLS) to select a subset of those SNPs.

We plan to study merits and demerits of these strategies and the computational issues associated with them in detail through synthetic studies as well as in the GWAS data from MCTFR. Finally, the current evaluation map based formulation requires the existence of an asymptotic distribution for the full model estimate. We plan to explore alternative formulation of evaluation maps under weaker conditions to bypass this, thus being able to tackle high-dimensional ($n < p$) situations.

It is important to remember that in a GWAS setting looking for causal factors of polygenic, quantitative traits is a complex problem. Small effect sizes and high amounts of LD—combined with the influence of environmental covariates—can make finding a set of SNPs behind that trait a noisy process. Typically the random effect error-term is earmarked to account for and quantify such heterogeneities at family-level, but how accurate this quantification is depends on the specific problem context. For this reason, in single-SNP models adjusting the p-values for FDR is important before selecting the final set of SNPs. While our proposed method is based on multi-SNP models, it may still need corrections to calibrate the potential of false discoveries. Finally, robustifying our proposed against data-level issues such as non-nuclear families, lack of individual-level data for some individuals warrant additional research. To this end, there is potential of adapting existing methods, such as Niu et al.^49^, to the paradigm of e-values.

## Supplementary Information

Supplementary Information.

## Data Availability

The genotype and phenotype data for the MCTFR sample used in this study are available through the Database of Genotypes and Phenotypes (dbGaP, phs000620.v1.p1). but restrictions apply to the availability of these data, were used under license for the current study, and so are not publicly available. Data are however available from corresponding author on responsible request.
