# Supplementary material for: Simultaneous selection of multiple important single nucleotide polymorphisms in familial genome wide association studies data
Source: Sci Rep. 2023 May 25;13:8476. doi: 10.1038/s41598-023-35379-y (PMC10213008; doi:10.1038/s41598-023-35379-y)
Supplement: Supplementary file 1 — Supplementary Information. [file 41598_2023_35379_MOESM1_ESM.pdf]

Simultaneous Selection of Multiple Important Single Nucleotide Polymorphisms in Familial Genome Wide Association Studies Data

Supplementary material

Appendix

A Theory of e-values

We present the details of our methodology in this Appendix. Sections A.1 and A.2 summarize the existing method of e-values that performs best subset variable selection in a wide range of statistical models[1]. We build on this framework and present new results for better detection of weak SNP signals. Section A.3 elaborates on the bootstrap implementation of this methodology, while Section A.4 presents proofs of all theoretical results.

A.1 Models and evaluation maps

In a general modelling situation where one needs to estimate a set of parameters $\theta \in \mathbb{R}^d$ from an array of samples $\mathscr{B}_n = \{B_1, \ldots, B_n\}$ at stage n, any hypothesis or statistical model corresponds to a subset of the full parameter space. Here we consider the model spaces $\Theta_m \subseteq \mathbb{R}^d$ in which some elements of the parameter vector have fixed values, while others are estimated from the data. Formally, a generic parameter vector $\theta_m \in \Theta_m$ consists of entries

$$\theta_{\mathscr{M}j} = \begin{cases} \text{Unknown } \theta_{\mathscr{M}j} & \text{for } j \in \mathscr{S}, \\ \text{Known } c_j & \text{for } j \notin \mathscr{S}. \end{cases}$$

for some $\mathscr{S} \subseteq \{1, \ldots, d\}$. Thus the estimable index set $\mathscr{S}$ and fixed elements $C = (c_j : j \notin \mathscr{S})$ fully specify any model $\mathscr{M}$ in this setup.

We obtain the full model estimates as minimizers of an estimating equation:

$$\hat{\theta} = \arg\min_{\theta} \Psi(\theta) = \arg\min_{\theta} \sum_{i=1}^{n} \psi_i(\theta, B_i). \tag{A.1}$$

The only condition we impose on these generic estimating functionals $\psi_i(\cdot)$ are:
(P1) The population version of (A.1) has a unique minimizer θ_0, i.e.

$$\theta_0 = \arg\min_{\theta} \mathbb{E} \sum_{i=1}^{n} \psi_i(\theta, B_i).$$

(P2) There exist a sequence of positive numbers $a_n \uparrow \infty$ and a p-dimensional probability distribution $\mathbb{T}_0$ such that $a_n(\hat{\theta} - \theta_0) \rightsquigarrow \mathbb{T}_0$.

We designate θ_0 as the *true parameter vector*, some elements of which are potentially set to 0. We can now classify any candidate model $\mathscr{M}$ into one of the two classes: the ones that satisfy $\theta_0 \in \Theta_{\mathscr{M}}$, and the ones that do not. We denote these two types of models by *adequate* and *inadequate models*, respectively. Given the data and unknown θ_0, we want to determine if a candidate model is adequate or inadequate.

For this we need coefficient estimates $\hat{\theta}_{\mathscr{M}}$ for model $\mathscr{M}$. We do so by just replacing elements of $\hat{\theta}$ not in $\mathscr{S}$ by corresponding elements of c. This means that for the jth element, $j = 1, \ldots, p$, we have

$$\hat{\theta}_{\mathscr{M}j} = \begin{cases} \text{Unknown } \hat{\theta}_j & \text{for } j \in \mathscr{S}, \\ \text{Known } c_j & \text{for } j \notin \mathscr{S}. \end{cases}$$

We denote the probability distribution of a random variable $\mathbf{T}$ by $[\mathbf{T}]$. With this notation, we aim to compare the above model estimate distributions with the full model distribution, i.e. $[\hat{\theta}_{\mathscr{M}}]$ with $[\hat{\theta}]$. For this we define an *evaluation map* function

$E : \mathbb{R}^d \times \tilde{\mathbb{R}}^d \to [0, \infty)$ that measures the relative position of $\hat{\theta}_{\mathscr{M}}$ with respect to $[\hat{\theta}]$. Here $\tilde{\mathbb{R}}^d$ is the set of probability measures on $\mathbb{R}^d$. We assume that E satisfies the following conditions:

(E1) For any probability distribution $\mathbb{G} \in \tilde{\mathbb{R}}^d$ and $x \in \mathbb{R}^d$, E is invariant under location and scale transformations:

$$E(x, \mathbb{G}) = E(ax + b, [a\mathbf{G} + b]); \quad a \in \mathbb{R} \neq 0, b \in \mathbb{R}^d.$$

where the random variable $\mathbf{G}$ has distribution $\mathbb{G}$.

(E2) The evaluation map E is lipschitz continuous under the first argument:

$$|E(x, \mathbb{G}) - E(y, \mathbb{G})| < \|x - y\|^\alpha; \quad x, y \in \mathbb{R}^d, \alpha > 0.$$

(E3) Suppose $\{\mathbb{Y}_n\}$ is a tight sequence of probability measures in $\tilde{\mathbb{R}}^d$ with weak limit $\mathbb{Y}_\infty$. Then $E(x, \mathbb{Y}_n)$ converges uniformly to $E(x, \mathbb{Y}_\infty)$.

(E4) Suppose $\mathbf{Z}_n$ is a sequence of random variables such that $\|\mathbf{Z}_n\| \xrightarrow{P} \infty$. Then $E(\mathbf{Z}_n, \mathbb{Y}_n) \xrightarrow{P} 0$.

For any $x \in \mathbb{R}^d$ and $[\mathbf{X}] \in \tilde{\mathbb{R}}^d$ with a positive definite covariance matrix $\mathbb{V}\mathbf{X}$, following are examples of the evaluations functions covered by the above set of conditions:

$$E_1(x, [\mathbf{X}]) = \left[1 + \left\| \frac{x - \mathbb{E}\mathbf{X}}{\sqrt{\mathrm{diag}(\mathbb{V}\mathbf{X})}} \right\|^2 \right]^{-1}; \quad E_2(x, [\mathbf{X}]) = \exp\left[- \left\| \frac{x - \mathbb{E}\mathbf{X}}{\sqrt{\mathrm{diag}(\mathbb{V}\mathbf{X})}} \right\| \right] \tag{A.2}$$

Data depths[2–4] also constitute a broad class of point-to-distribution proximity functions that satisfy the above regularity conditions for evaluation maps. Indeed, Majumdar and Chatterjee[1] used Mahalanobis depth and halfspace depth[2] as evaluation function to perform model selection. However, the conditions (E1) and (E4) are weaker than those imposed on a traditional depth function[3]. Conditions (E2) and (E3) are not required of depth functions in general, but they arise implicitly in several implementations of data depth[5]. The theoretical results we state here are based on a general evaluation map and not depth functions. To emphasize this point, in this paper we use the non-depth evaluation functions E_1 and E_2 as in (A.2) above.

A.2 Model selection using e-values

Depending on the choice of the data sequence $\mathscr{B}_n$, $E(\hat{\theta}_{\mathscr{M}}, [\hat{\theta}])$ can take different values. For any candidate model $\mathscr{M}$, we denote the distribution of the corresponding evaluation map—across all possible random data sequences $\mathscr{B}_n$—by $\mathbb{E}_{\mathscr{M}n}$. For simplicity we drop the n in its subscript, i.e. $\mathbb{E}_{\mathscr{M}n} \equiv \mathbb{E}_{\mathscr{M}}$. These distributions are informative of the behavior of parameter estimates $\hat{\theta}_{\mathscr{M}}$. We use them as a tool to distinguish between inadequate and adequate models. Given a single set of samples, we use resampling schemes that satisfy standard regularity conditions[1] to get consistent approximations of $\mathbb{E}_{\mathscr{M}}$.

We now define the **e-value** to compare the different model estimates and eventually perform selection of important SNPs from a multi-SNP model. Loosely construed, any functional of the evaluation map distribution $\mathbb{E}_{\mathscr{M}}$ that can act as model evidence is an e-value. For example, Majumdar and Chatterjee[1] took the mean functional of $\mathbb{E}_{\mathscr{M}}$ (say $\mu(\mathbb{E}_{\mathscr{M}})$) as e-value, and proved the following result (see Theorem 5.1 therein):

Theorem A.1. *Consider estimators satisfying conditions (P1) and (P2), and an evaluation map E satisfying the conditions (E1), (E2) and (E4). Also suppose that*

$$\lim_{n \to \infty} \mu(\mathbb{Y}_n) = \mu(\mathbb{Y}_\infty) < \infty,$$

for any tight sequence of probability measures $\{\mathbb{Y}_n\}$ in $\tilde{\mathbb{R}}^d$ with weak limit $\mathbb{Y}_\infty$. Then as $n \to \infty$,

- *For the full model, $\mu(\mathbb{E}_*) \to \mu_\infty$ for some $0 < \mu_\infty < \infty$;*
- *For any adequate model, $|\mu(\mathbb{E}_{\mathscr{M}}) - \mu(\mathbb{E}_*)| \to 0$,*
- *For any inadequate model, $\mu(\mathbb{E}_{\mathscr{M}}) \to 0$.*

Taking data depths as evaluation functions leads to a further result that $\mu(\mathbb{E}_*) < \mu(\mathbb{E}_{\mathscr{M}})$ for any adequate model $\mathscr{M}$ and large enough n.

A similar result holds for tail quantiles as well. Denote the qth population quantile of $\mathbb{E}_{\mathscr{M}}$ by $c_q(\mathbb{E}_{\mathscr{M}})$. Then we have equivalent results to Theorem A.1 as $n \to \infty$:

Theorem A.2. *Given that the estimator $\hat{\theta}$ satisfies conditions (P1) and (P2), and the evaluation map satisfies conditions (E1)-(E4), we have*

$$c_q(\mathbb{E}_*) \to c_{q,\infty} < \infty, \tag{A.3}$$
$$|c_q(\mathbb{E}_{\mathcal{M}}) - c_q(\mathbb{E}_*)| \to 0 \text{ when } \mathcal{M} \text{ is adequate}, \tag{A.4}$$
$$c_q(\mathbb{E}_{\mathcal{M}}) \to 0 \text{ when } \mathcal{M} \text{ is inadequate}. \tag{A.5}$$

A one-step model selection procedure equivalent to Algorithm 1 follows when we restrict our model selection procedure to the restricted model class

$$\mathbb{M}_0 = \{\mathcal{M} \equiv (\mathcal{S}, C) : c_j = 0 \quad \forall \quad j \notin \mathcal{S}\}.$$

In other words, we only consider models that have known parameters set at 0. In this scenario, non-zero indices of θ_0 (say $\mathcal{S}_0$) can be recovered through Algorithm 1. This follows from applying Corollary 5.3 in[1] in our setup, with mean e-values replaced by quantile e-values.

A.3 Bootstrap procedure

We use Generalized Bootstrap (GBS)[6] to obtain approximations of the sampling distributions $\mathbb{E}_{-j}$ and $\mathbb{E}_*$. It calculates bootstrap equivalents of the parameter estimate $\hat{\theta}$ by minimizing a version of the estimating equation in (A.1) with random weights:

$$\hat{\theta}_r = \arg\min_{\theta} \sum_{i=1}^{n} \mathbb{W}_i \psi_i(\theta, B_i). \tag{A.6}$$

The resampling weights $(\mathbb{W}_1, \ldots, \mathbb{W}_n)$ are non-negative exchangeable random variables chosen independent of the data, and satisfy the following conditions:

$$\mathbb{E}\mathbb{W}_1 = 1; \quad \mathbb{V}\mathbb{W}_1 = \tau_n^2 \uparrow \infty; \quad \tau_n^2 = o(a_n^2) \tag{A.7}$$
$$\mathbb{E}W_1 W_2 = O(n^{-1}); \quad \mathbb{E}W_1^2 W_2^2 \to 1; \quad \mathbb{E}W_1^4 < \infty \tag{A.8}$$

with $W_i := (\mathbb{W}_i - 1)/\tau_n; i = 1, \ldots, n$ being the centered and scaled resampling weights. Under standard regularity conditions on the estimating functional $\Psi(\cdot)$[1,6] and conditional on the data, $(a_n/\tau_n)(\hat{\theta}_w - \hat{\theta})$ converges to the same asymptotic distribution as $a_n(\hat{\theta} - \theta_0)$, i.e. $\mathbb{T}_0$.

We use empirical quantiles of the full model bootstrap samples as the quantile e-value estimates. Specifically, we go through the following steps:

- Fix $q, t \in (0, 1)$;
- Generate two independent set of bootstrap weights, of size R and R_1, and obtain the corresponding approximations to the full model sampling distribution, say $[\hat{\theta}_r]$ and $[\hat{\theta}_{r_1}]$;
- For $j = 1, 2, \ldots p$ and estimate the e-value of the jth predictor as the empirical q^{th} quantile of $\hat{\mathbb{E}}_{-j} := [E(\hat{\theta}_{r,-j}, [\hat{\theta}_{r_1}])]$, with $\hat{\theta}_{r,-j}$ obtained from $\hat{\theta}_r$ by replacing the jth coordinate with 0;
- Estimate the set of non-zero covariates as $\hat{\mathcal{S}}_0 = \{j : c_q(\hat{\mathbb{E}}_{-j}) < c_{qt}(\hat{\mathbb{E}}_*)\}$.

Conditions (A.7) and (A.8) on the resampling weights ensure bootstrap-consistent approximation of the evaluation map quantiles:

Theorem A.3. *Given the estimator $\hat{\theta}$ and evaluation map E in Theorem A.2, and a generalized bootstrap scheme satisfying (A.7) and (A.8), we get*

$$|c_q(\hat{\mathbb{E}}_{\mathcal{M}}) - c_q(\hat{\mathbb{E}}_*)| \stackrel{P_n}{\to} o_P(1) \text{ when } \mathcal{M} \text{ is adequate}, \tag{A.9}$$
$$c_q(\hat{\mathbb{E}}_{\mathcal{M}}) \stackrel{P_n}{\to} o_P(1) \text{ when } \mathcal{M} \text{ is inadequate}. \tag{A.10}$$

where P_n is probability conditional on the data.

Generalized bootstrap covers a large array of resampling procedures, for example the m-out-of-n bootstrap and a scale-enhanced version of the bayesian bootstrap. Furthermore, given that $\psi_i(.)$ are twice differentiable in a neighborhood of θ_0 and some other conditions in[6], there is an approximate representation of $\hat{\theta}_w$:

$$\hat{\theta}_r = \hat{\theta} - \frac{\tau_n}{a_n}\left[\sum_{i=1}^{n} W_i \psi_i''(\hat{\theta}, B_i)\right]^{-1} \sum_{i=1}^{n} W_i \psi_i'(\hat{\theta}, B_i) + \mathbf{R}_{wn}. \tag{A.11}$$

with $\mathbb{E}_w \|\mathbf{R}_{wn}\|^2 = o_P(1)$.

Given the full model estimate $\hat{\theta}$, and the score vectors $\psi_i'(\hat{\theta}, B_i)$ and hessian matrices $\psi_i''(\hat{\theta}, B_i)$, (A.11) allows us to obtain multiple copies of $\hat{\theta}_r$ through Monte-Carlo simulation of several arrays of bootstrap weights. This bypasses the need to fit the full model for each bootstrap sample, resulting in extremely fast computation of e-values.

Recall from the main paper our Linear Mixed Model (LMM) setup. Suppose there are m families in total, with the ith pedigree containing n_i individuals. Denote by $y_i = (y_{i1}, \ldots, y_{in_i})^T$ the quantitative trait values for individuals in that pedigree, while the matrix $\mathbf{G}_i \in \mathbb{R}^{n_i \times p_g}$ contains their genotypes for a number of SNPs. Let $\mathbf{C}_i \in \mathbb{R}^{n_i \times p}$ denote the data on p covariates for individuals in the pedigree i. Given these, we consider the following model.

$$\mathbf{Y}_i = \alpha + \mathbf{G}_i \beta_g + \mathbf{C}_i \beta_c + \varepsilon_i, \tag{A.12}$$

with α the intercept term, β_g and β_c fixed coefficient terms corresponding to the multiple SNPs and covariates, respectively, and $\varepsilon_i \sim \mathscr{N}_{n_i}(\mathbf{0}, \mathbf{V}_i)$ the random error term. To account for the within-family dependency structure, we break up the random error variance into three independent components:

$$\mathbf{V}_i = \sigma_a^2 \Phi_i + \sigma_c^2 \mathbf{1}\mathbf{1}^T + \sigma_e^2 \mathbf{I}_{n_i}. \tag{A.13}$$

The three variance components $\sigma_a^2 \Phi_i, \sigma_c^2 \mathbf{1}\mathbf{1}^T$, and $\sigma_e^2 \mathbf{I}_{n_i}$ represent polygenic effects, shared environmental variations, and random variations, respectively.

We adapt the approximation of (A.11) to the LMM in (A.12). We first obtain the maximum likelihood estimates $\hat{\beta}_g, \hat{\sigma}_a^2, \hat{\sigma}_c^2, \hat{\sigma}_e^2$ through fitting the LMM. Then we replace the variance components in (A.13) with corresponding estimates to get $\hat{\mathbf{V}}_i$ for i^{th} pedigree, and aggregate them to get the covariance matrix estimate for all samples:

$$\hat{\mathbf{V}} = \text{diag}(\hat{\mathbf{V}}_1, \ldots, \hat{\mathbf{V}}_m).$$

We take m random draws from Gamma$(1,1) - 1$, say $\{w_{r1}, \ldots, w_{rm}\}$, as resampling weights in (A.11), using the same weight for all members of a pedigree. Consequently, the bootstrapped coefficient estimate $\hat{\beta}_{rg}$ has the following representation:

$$\hat{\beta}_{rg} \simeq \hat{\beta}_g + \frac{\tau_n}{\sqrt{n}} (\mathbf{G}^T \hat{\mathbf{V}}^{-1} \mathbf{G})^{-1} \mathbf{W}_r \mathbf{G}^T \hat{\mathbf{V}}^{-1} (y - \mathbf{G}\hat{\beta}_g). \tag{A.14}$$

with $\mathbf{G} = (\mathbf{G}_1^T, \ldots, \mathbf{G}_m^T)^T$ and $\mathbf{W}_r = \text{diag}(w_{r1}\mathbf{I}_4, \ldots, w_{rm}\mathbf{I}_4)$. Finally we repeat the procedure for two independent sets of resampling weights, say of sizes R and R_1, to obtain two collections of bootstrapped estimates $\{\hat{\beta}_{1g}, \ldots, \hat{\beta}_{Rg}\}$.

A.4 Proof of theoretical results

Proof of Theorem A.2. Define $c_{q,\infty} = q^{\text{th}}$ quantile of $\mathbb{T}_0$. Now following assumption (E1),

$$\begin{aligned} c_q(\mathbb{E}_*) &= \inf_\theta \{E(\theta, [\hat{\theta}]) : \mathbb{F}_* \geq q\} \\ &= \inf_\theta \{E(a_n(\theta - \theta_0), [a_n(\hat{\theta} - \theta_0)]) : a_n(\mathbb{F}_* - \theta_0) \geq q\}. \end{aligned}$$

where $\mathbb{F}_*$ is the probability distribution function of $E(\hat{\theta}, [\hat{\theta}])$. Part 1 is proved following assumptions (P2) and (E3).

Now if $\mathscr{M}$ is adequate, following assumption (E1),

$$E(\hat{\theta}_{\mathscr{M}}, [\hat{\theta}]) = E(\hat{\theta}_{\mathscr{M}} - \theta_0, [\hat{\theta} - \theta_0]). \tag{A.15}$$

Decompose the first argument as

$$\hat{\theta}_{\mathscr{M}} - \theta = (\hat{\theta}_{\mathscr{M}} - \hat{\theta}) + (\hat{\theta} - \theta_0). \tag{A.16}$$

By definition, $\hat{\theta}_{\mathscr{M}j} - \hat{\theta}_j = 0$ if $j \in \mathscr{S}$, else equals $\theta_{0j} - \hat{\theta}_j$. Thus for the first summand in (A.16) we have

$$\hat{\theta}_{\mathscr{M}} - \hat{\theta} = O_P(1/a_n).$$

Going back to (A.15), this implies

$$|E(\hat{\theta}_{\mathscr{M}} - \theta_0, [\hat{\theta} - \theta_0]) - E(\hat{\theta} - \theta_0, [\hat{\theta} - \theta_0])| < O_P(a_n^{-\alpha}),$$

using lipschitz continuity in assumption (E2), i.e

$$|E(\hat{\theta}_m, [\hat{\theta}]) - E(\hat{\theta}, [\hat{\theta}])| < O_P(a_n^{-\alpha}),$$

again using (E1). Part 2 now follows.

For part 3, we apply (E1) to get

$$E(\hat{\theta}_m, [\hat{\theta}]) = E(a_n(\hat{\theta}_{\mathscr{M}} - \theta_0), [a_n(\hat{\theta} - \theta_0)]). \quad (A.17)$$

We then decompose the first argument as

$$a_n(\hat{\theta}_{\mathscr{M}} - \theta_0) = a_n(\hat{\theta}_{\mathscr{M}} - \theta_{\mathscr{M}}) + a_n(\theta_{\mathscr{M}} - \theta_0) \quad (A.18)$$

Since $\mathscr{M}$ is inadequate, $\theta_{\mathscr{M}j} \neq \theta_{0j}$ when $j \notin \mathscr{S}$. So $\|a_n(\theta_{\mathscr{M}} - \theta_0)\| \uparrow \infty$ as $a_n \uparrow \infty$. Applying (E4) now proves part 3. □

Proof of Theorem A.3. The proof is fairly similar to that of Theorem 5.1 in Majumdar and Chatterjee[1], so we give a sketch. For the full model, the bootstrap is consistent, i.e. $a_n(\hat{\theta}_* - \theta_0)$ and $(a_n/\tau_n)(\hat{\theta}_{r*} - \hat{\theta}_*)$ converge to same weak limit in probability, following theorems 2.2 and 2.3 in Majumdar and Chatterjee[1]. Specifically, conditions (A1)-(A6) in Majumdar and Chatterjee[1] ensure condition (P2) in our paper through theorem 2.2 therein, following which theorem 2.3 ensures that when (A1)-(A6) are satisfied, bootstrap consistency holds. The definition of $\hat{\theta}_{\mathscr{M}}$ now means that $a_n(\hat{\theta}_{\mathscr{M}} - \theta_{\mathscr{M}})$ and $(a_n/\tau_n)(\hat{\theta}_{r\mathscr{M}} - \hat{\theta}_{\mathscr{M}})$ converge to the same weak limit in probability for any model $\mathscr{M}$. A similar approach as the proof of parts 2 and 3 of theorem 5.1 now follows, with an additional term corresponding to bootstrap estimates in (A.16) and (A.18). □

B Discussion on gene-specific findings in the MCTFR data

GABRA2: As seen in the plots, the first two SNPs detected are close to two separate exons. The 4th and 5th detected SNPs, rs1808851 and rs279856, are at perfect LD with rs279858 in the larger 7188-individual dataset[7]. This SNP had not been genotyped in our sample, but is the marker in GABRA2 that is most frequently associated in the literature with alcohol abuse[8]. Interestingly, a single SNP RFGLS analysis of the same twin studies data that used Bonferroni correction on marginal p-values to detect SNPs had missed these SNPs[7]. This highlights the advantage of our approach.

ADH genes: Multiple studies have associated rs1229984 in the ADH1B gene (position 99318162 of chromosome 4) with alcohol dependence (https://www.snpedia.com/index.php/Rs1229984), which as seen in the plot of ADH2 is close to an exon region. Our data does not contain this marker, but detects one SNP 20 kb upstream of this, rs17027523. Another SNP, rs3775540 at position 99304544 has an e-value of 0.226, so narrowly misses detection. This is close to rs1229984, and also rs1042026 at position 99307309, which[9] found to be strongly associated with alcohol consumption.

The SNP rs17027523 is interesting: it resides in the uncharacterized long non-coding RNA gene LOC100507053. One previous study[10,11] found significant associations for 5 SNPs in this gene with alcohol consumption for African American population through single-SNP analysis on non-familial GWAS data. Notably, their analysis found a much stronger evidence of the association in African-American part of the sample than the European American part, while our findings are entirely from a Caucasian sample.

OPRM1: Many of the SNPs analyzed in this gene have very low e-values, and tend to cluster together. The minor allele of the SNP rs1799971 (chr 6, position 154039662) has been associated with stronger alcohol cravings (https://www.snpedia.com/index.php/Rs1799971), and we detect rs12662873 at position 154040810.

CYP2E1: Five of the 9 SNPs studied are detected through our analysis. Four of them are within 10 kb of one another (base pairs 133534822 to 133543210 in chr 10). In the analysis of[12] rs4646976 at 133534223 position was most associated with a measure of breath alcohol concentration: this is within our detected region. This study had also detected rs4838767 in the promoter region of CYP2E1 (position 133520114) associated with multiple alcohol consumption measures. We detect rs9419702 at position 133531153.

ALDH2: All 6 SNPs we study are close to exons, and 5 get picked up by the e-value procedure. While all five are at a lesser base pair position than the well-known SNP rs671 (https://www.snpedia.com/index.php/Rs671, position 111803962), one of the SNPs we analyze (rs16941437) is within 10 kb upstream of this SNP.

COMT: The SNP rs4680 has long been associated with schizophrenia and substance abuse, including alcoholism. A case-control study[13] associated rs4680 and rs165774 with alcohol dependence through a SNP-wise chi-squared test, and had these two SNPs in high LD in their study population. Compared to this, in our simultaneous model of all COMT polymorphisms, the more well-known rs4680 has a below threshold *e*-value.

SLC6A3: Our analysis does not detect rs27072, which has been associated with alcohol withdrawal symptoms (https://www.snpedia.com/index.php/Rs27072).

Finally, most *e*-values for the last 3 genes, i.e. SLC6A3, SLC6A4 and DRD2, are large: indicating weak SNP signals. We found this observation interesting, because variants of these genes have known interaction effects behind alcohol withdrawal-induced seizure[14] and bipolar disorder[15], as well as additive effect on the susceptibility to smoking addiction[16].

C Outputs for MCTFR data analysis

Each table gives the 90th percentile *e*-values, which are plotted in figures 2, 3, and 4 in main paper, of SNPs analyzed in the gene. Column 'Association' is obtained from the sign of the SNP coefficient in the full model.

SNP name	Location	*e*-value	Association
rs16859227	46250605	0.89	+
rs572227	46251393	0.13	-
rs534459	46256805	0.24	+
rs2119183	46272806	0.92	-
rs502038	46280318	0.58	+
rs1808851	46311447	0.00	+
rs279856	46317923	0.00	-
rs3775282	46321863	0.86	-
rs279841	46340763	0.75	+
rs10805145	46358331	0.73	-
rs13152740	46381221	0.86	-

Table C.1. SNPs for GABRA2, chr4, position 46243548 - 46390039; *e*-value cutoff 0.72

References

1. Majumdar, S. & Chatterjee, S. Feature selection using e-values. In *Proceedings of ICML* (2022).
2. Tukey, J. Mathematics and picturing data. In James, R. (ed.) *Proceedings of the International Congress on Mathematics*, vol. 2, 523–531 (1975).
3. Zuo, Y. & Serfling, R. General notions of statistical depth functions. *Ann. Stat.* **28-2**, 461–482 (2000).
4. Zuo, Y. Projection-based depth functions and associated medians. *Ann. Stat.* **31**, 1460–1490 (2003).
5. Mosler, K. Depth statistics. In Becker, C., Fried, R. & Kuhnt, S. (eds.) *Robustness and Complex Data Structures*, 17–34 (Springer Berlin Heidelberg, 2013).
6. Chatterjee, S. & Bose, A. Generalized bootstrap for estimating equations. *Ann. Stat.* **33**, 414–436 (2005).
7. Irons, D. E. *Characterizing specific genetic and environmental influences on alcohol use*. Ph.D. thesis, University of Minnesota (2012).
8. Cui, W. Y., Seneviratne, C., Gu, J. & Li, M. D. Genetics of GABAergic signaling in nicotine and alcohol dependence. *Hum. Genet.* **131**, 843–855 (2012).
9. Macgregor, S., Lind, P. A., Bucholtz, K. K. *et al.* Associations of adh and aldh2 gene variation with self report alcohol reactions, consumption and dependence: an integrated analysis. *Hum. Mol. Genet.* **18**, 580–593 (2008).
10. Gelernter, J., Kranzler, H. R., Sherva, R., Almasy, L. *et al.* Genome-wide association study of alcohol dependence: significant findings in African- and European-Americans including novel risk loci. *Mol. Psychiatry* **19**, 41–49 (2014).
11. Xu, K., Kranzler, H. R., Sherva, R., Sartor, C. E. *et al.* Genomewide Association Study for Maximum Number of Alcoholic Drinks in European Americans and African Americans. *Alcohol Clin. Exp. Res.* **39**, 1137–1147 (2015).

SNP name	Location	e-value	Association
rs17027299	99078105	0.84	-
rs9307222	99101051	0.76	-
rs10006414	99101401	0.49	+
rs9994641	99101605	0.48	+
rs13134014	99104879	0.75	-
rs6820691	99105055	0.76	+
rs6820913	99125659	0.81	+
rs6532729	99146436	0.67	-
rs13150538	99152631	0.49	-
rs17027380	99157450	0.63	-
rs17494998	99160699	0.41	+
rs549467	99172232	0.81	+
rs2034677	99187874	0.62	+
rs12508445	99190653	0.01	-
rs10003496	99197839	0.81	+
rs10005811	99208603	0.02	+
rs603215	99214851	0.78	-
rs433146	99229839	0.87	-
rs17027456	99235747	0.31	-
rs17561798	99235941	0.85	+
rs10516428	99237439	0.45	-
rs6532731	99251006	0.80	+
rs7694221	99260423	0.90	+
rs10028330	99268949	0.70	-
rs10022047	99296818	0.49	+
rs17027523	99298979	0.05	+
rs17027530	99303633	0.69	+
rs3775540	99304544	0.23	-
rs3756088	99309404	0.89	-
rs13103626	99317251	0.75	+
rs10516430	99337881	0.62	+
rs9884594	99359318	0.68	-
rs12503056	99369061	0.63	+
rs2004316	99381148	0.43	-
rs4303985	99399748	0.87	-
rs4414961	99403784	0.86	-
rs12509267	99407299	0.80	+
rs6838913	99408106	0.84	-
rs4374629	99411783	0.85	+
rs4527483	99421741	0.89	+
rs10009693	99423280	0.90	-
rs10023791	99425353	0.88	+
rs955931	99428163	0.88	-
rs17027628	99428608	0.85	-

Table C.2. SNPs for ADH genes, chr4, position 99070977 - 99435737; e-value cutoff 0.225

12. Lind, P. A., Macgregor, S., Heath, A. C. & Madden, P. A. F. Association between *in vivo* alcohol metabolism and genetic variation in pathways that metabolize the carbon skeleton of ethanol and NADH reoxidation in the Alcohol Challenge Twin Study. *Alcohol Clin. Exp. Res.* **36**, 2074–2085 (2012).

13. Voisey, J., Swagell, C. D., Hughes, I. P. *et al.* A novel SNP in COMT is associated with alcohol dependence but not opiate or nicotine dependence: a case control study. *Behav. Brain Funct.* **7** (2011).

14. Karpyak, V. M., Biernacka, J. M., Weg, M. W. *et al.* Interaction of SLC6A4 and DRD2 polymorphisms is associated with a

history of delirium tremens. *Addict. Biol.* **15**, 23–34 (2010).

15. Wang, T. Y., Lee, S. Y., Chen, S. L. *et al.* Gender-specific association of the SLC6A4 and DRD2 gene variants in bipolar disorder. *Int. J. Neuropsychopharmacol.* **17**, 211–222 (2014).

16. Erblich, J. A., Lerman, C., Self, D. W. *et al.* Effects of dopamine d2 receptor (drd2) and transporter (slc6a3) polymorphisms on smoking cue-induced cigarette craving among african-american smokers. *Mol. Psychiatry* **10**, 407–414 (2005).

SNP name	Location	*e*-value	Association
rs2000371	154011024	0.39	-
rs9371718	154011615	0.08	-
rs12211203	154016936	0.63	-
rs1937600	154017197	0.02	-
rs9397637	154022718	0.00	+
rs1937590	154036895	0.63	+
rs12662873	154040810	0.18	+
rs12661209	154044112	0.84	-
rs1316368	154055754	0.00	-
rs1937587	154060023	0.27	-
rs6921403	154063906	0.00	-
rs1937580	154076643	0.00	+
rs1937645	154082228	0.00	+
rs1892361	154099619	0.00	-
rs1937633	154104857	0.04	-
rs1937631	154105011	0.00	-
rs12527197	154107836	0.02	+
rs1892360	154111701	0.74	-
rs1892359	154112042	0.65	-
rs1892356	154112263	0.56	+
rs1937622	154113139	0.54	-
rs10485258	154113409	0.72	-
rs1937619	154114583	0.58	-
rs1748289	154121980	0.77	-
rs1781619	154135968	0.64	-
rs652051	154139344	0.74	+
rs10485262	154140199	0.69	-
rs9371312	154145492	0.81	+
rs1332849	154151117	0.48	-
rs9371749	154153369	0.28	+
rs9285539	154154532	0.08	+
rs9322439	154156250	0.07	+
rs11752884	154159710	0.25	-
rs4869813	154173845	0.13	+
rs4870241	154174963	0.00	-
rs9384156	154186720	0.13	+
rs2065139	154192175	0.89	-
rs689219	154198820	0.00	-
rs9371761	154202578	0.20	-
rs12199858	154204327	0.00	+
rs9371762	154213973	0.00	-
rs612450	154214357	0.00	-
rs9384159	154219177	0.00	+
rs6938958	154220427	0.00	-
rs581564	154221214	0.00	+
rs12202611	154237443	0.76	-
rs4870255	154237937	0.88	-

Table C.3. SNPs for OPRM1, chr6, position 154010496 - 154246867; *e*-value cutoff 0.225

SNP name	Location	*e*-value	Association
rs10872828	133525348	0.72	-
rs9419702	133531153	0.09	-
rs7083395	133532269	0.77	+
rs9419624	133534822	0.06	+
rs7906770	133536902	0.28	-
rs9419569	133541881	0.06	+
rs9419629	133543210	0.06	+
rs7093241	133556596	0.72	-
rs9419649	133561098	0.91	-

Table C.4. SNPs for CYP2E1, chr10, position 133520406 - 133561220; *e*-value cutoff 0.72

SNP name	Location	*e*-value	Association
rs7398343	111774068	0.34	-
rs7297186	111778178	0.36	+
rs3803167	111785586	0.00	+
rs10219736	111788402	0.00	-
rs16941437	111793039	0.00	-
rs3742004	111798553	0.75	+

Table C.5. SNPs for ALDH2, chr12, position 111766887 - 111817529; *e*-value cutoff 0.72

SNP name	Location	*e*-value	Association
rs4646312	19948337	0.41	-
rs165656	19948863	0.22	-
rs165722	19949013	0.24	+
rs2239393	19950428	0.50	+
rs4680	19951271	0.60	+
rs4646316	19952132	0.81	-
rs165774	19952561	0.72	-
rs174699	19954458	0.07	+
rs165599	19956781	0.58	-
rs165728	19957023	0.02	-
rs165815	19959473	0.00	+
rs5993891	19959746	0.04	-
rs887199	19961955	0.04	-
rs2239395	19962203	0.07	+
rs2518824	19962963	0.59	+

Table C.6. SNPs for COMT, chr22, position 19941607 - 19969975; *e*-value cutoff 0.72

SNP name	Location	*e*-value	Association
rs27072	1394522	0.87	+
rs40184	1395077	0.78	-
rs11564771	1398797	0.80	-
rs11133767	1401580	0.79	+
rs6869645	1404548	0.82	+
rs3776512	1407116	0.84	+
rs6347	1411412	0.83	-
rs27048	1412645	0.90	-
rs2042449	1416646	0.63	+
rs13161905	1417212	0.72	-
rs2735917	1420268	0.92	+
rs464049	1423905	0.21	-
rs460700	1429969	0.00	-
rs460000	1432825	0.00	+
rs4975646	1433401	0.88	-
rs403636	1438354	0.78	-
rs2617605	1442521	0.89	+
rs6350	1443199	0.93	+

Table C.7. SNPs for SLC6A3, chr5, position 1392790 - 1445430; *e*-value cutoff 0.72

SNP name	Location	*e*-value	Association
rs16967029	30195292	0.79	+
rs2051810	30195841	0.84	-
rs11658318	30206059	0.72	-
rs8079471	30218317	0.64	+
rs3760454	30222002	0.90	+

Table C.8. SNPs for SLC6A4, chr17, position 30194319 - 30236002; *e*-value cutoff 0.63

SNP name	Location	*e*-value	Association
rs2514229	113410000	0.87	-
rs11214654	113410917	0.86	+
rs7937641	113415976	0.63	-
rs12222458	113417603	0.73	-
rs10736470	113418371	0.73	-
rs12576506	113419869	0.85	+
rs10750025	113424042	0.66	+
rs7952106	113424558	0.70	-
rs4373974	113430486	0.88	-
rs4130345	113436487	0.88	-
rs7123697	113440331	0.78	+
rs6589386	113443753	0.75	+
rs4132966	113451589	0.86	+
rs7940164	113451765	0.90	-
rs4245155	113457324	0.92	-
rs11607834	113461680	0.92	-
rs12280220	113469219	0.93	-

Table C.9. SNPs for DRD2, chr11, position 113409595 - 113475691; *e*-value cutoff 0.63
